# The role of cysteine residues in the allosteric modulation of the chromophore phototransformations of biphotochromic fluorescent protein SAASoti

**DOI:** 10.1038/s41598-021-03634-9

**Published:** 2021-12-21

**Authors:** A. V. Gavshina, N. K. Marynich, M. G. Khrenova, I. D. Solovyev, A. P. Savitsky

**Affiliations:** 1 A.N. Bach Institute of Biochemistry, Research Center of Biotechnology of the Russian Academy of Sciences, Moscow, Russia; 2 Department of Chemistry, Lomonosov Moscow State University, Moscow, Russia

**Keywords:** Biological fluorescence, Computational biophysics, Chemical modification, Biophysical chemistry

## Abstract

Biphotochromic fluorescent protein SAASoti contains five cysteine residues in its sequence and a V127T point mutation transforms it to the monomeric form, mSAASoti. These cysteine residues are located far from the chromophore and might control its properties only allosterically. The influence of individual, double and triple cysteine substitutions of mSAASoti on fluorescent parameters and phototransformation reactions (irreversible green-to-red photoconversion and reversible photoswitching) is studied. A set of mSAASoti mutant forms (C21N, C117S, C71V, C105V, C175A, C21N/C71V, C21N/C175A, C21N/C71G/C175A) is obtained by site-directed mutagenesis. We demonstrate that the C21N variant exists in a monomeric form up to high concentrations, the C71V substitution accelerates photoconversion to the red form and the C105V variant has the maximum photoswitching rate. All C175A-containing variants demonstrate different photoswitching kinetics and decreased photostability during subsequent switching cycles compared with other considered systems. Classical molecular dynamic simulations reveal that the F177 side chain located in the vicinity of the chromophore is considerably more flexible in the mSAASoti compared with its C175A variant. This might be the explanation of the experimentally observed slowdown the thermal relaxation rate, i.e., trans–cis isomerization of the chromophore in mSAASoti upon C175A substitution.

## Introduction

Phototransformable fluorescent proteins (PTFPs) are essential components when performing super-resolution techniques^1–3^. Such fluorescent markers must meet special requirements: (1) monomeric state for correct localization with the fusion protein; (2) chemical inertness of the surface protein groups. At the same time cysteine residues can participate in redox-processes including intra- and intermolecular disulfide bridges formation and sulfoxidation. Reactivity of cysteine residues can also restrict the application of some FPs in oxidative compartments (ER, bacteria periplasm)^4^. There is also an idea that cysteine residues can form intermolecular dimers before the correct β-barrel structure folds resulting in protein misfolding and undesirable aggregation^5^.

Reversible formation of intermolecular and internal disulfide bonds is one of the mechanisms of protein function regulation in vivo. In many cases a Cys-containing tripeptide glutathione forms a redox couple of reduced and oxidized form that is one of the key parameters in the living cell. It is known for some microorganisms, e.g., *E. coli*, glutathionylation of methionine synthase^6^ and PAPS reductase^7^ can occur during oxidative stress, resulting in inactivation of both enzymes. In mammalian organisms, SH-containing proteins can affect the formation of the cytoskeleton. Thus, Cys328 glutathionylation of vimentin inhibits filament elongation^8^. On the other hand, SH-containing proteins can affect thioredoxin and peroxiredoxin functions^9^. Such processes can be enhanced during oxidative stress, when the ratio of reduced/oxidized glutathione may decrease from 100/1 to 1/1^10^.

Consequently, on the one hand, amino acid residues with a reactive (including photoreactive) side chains (e.g., Cys) in the primary structure of fluorescent proteins limit their extensive use as fluorescent markers, but on the other hand, cysteine-free variants of several fluorescent proteins often demonstrate significant photostability loss and decreased brightness^5,11,12^. Interestingly, it is S146C substitution in the chromophore microenvironment of the mKate FP that resulted in the 12-fold more photostable variant named mStable due to sulfoxidation under illumination^13^. The smallest number of cysteine residues in the primary structure is characteristic of DsRed-family, the wild type gene contains only one cysteine residue^14^, whereas the monomerized variant—mRFP1^15^—and its derivatives (including mCherry) do not have any cysteine at all^16^. That is the reason why red FPs easily mature both in bacteria periplasm^17^ and in ER of eukaryotes^4^.

There are only two cysteine residues in EGFP sequence—C48 and C70—with SH-groups facing inside the β-barrel. First attempts to get cysteine-free secreted GFP variants were not successful, as the variant with double cysteine substitution lost its fluorescence, while single point mutants were resistant to dimerization before total folding, and their secretory pathway required extra stimulation^18^. Later obtained C48S/C70M variant of SGFP2 (GFP variant with extra brightness) successfully folds in oxidative conditions^12^. This approach demonstrates that rational site-directed mutagenesis sometimes comes with uncontrollable and undesirable changes of other fluorescent properties. As an alternative, the authors^4^ suggest using superfolder GFP variant (sfGFP) coupled with an optimized extravasation signal instead of cysteine substitution, as the protein folding rate in this case exceeds the rate of undesirable bonds formation between the proteins.

Because cysteine residues play significant role in function of fluorescent proteins as sensors the main goal of this research is to study the role of certain cysteine residues in photophysical and photochemical properties of biphotochromic fluorescent protein SAASoti.

## Results

**Figure 1:**
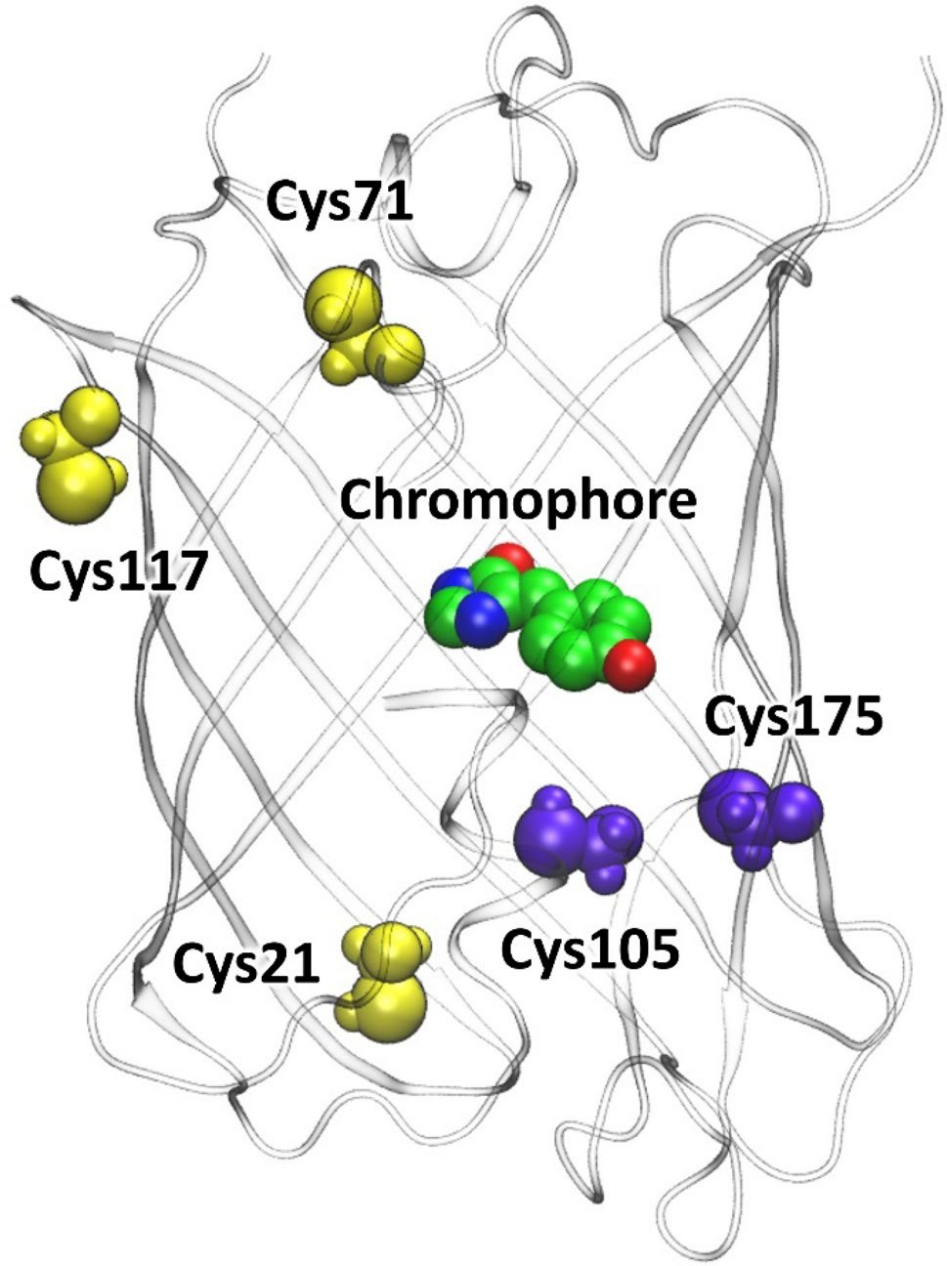
The calculated 3D structure of SAASoti fluorescent protein. The chromophore is colored by atom type (here and on the next figures carbon is green, oxygen—red, nitrogen—blue and hydrogen—white). The cysteine residues mutated in this study are colored in yellow if the side chain is oriented to the solution and in violet if the side chain is inside the β-barrel.

SAASoti FP was isolated from *Stylocoeniella armata* coral as a green-to-red photoconvertible fluorescent protein^19^. During the study on its physicochemical properties, SAASoti turned out to be reversibly photoswitchable between its green fluorescent and dark forms^20^. Subsequently, we also determined conditions when the red SAASoti form can undergo reversible photoswitching^21^. Significantly, its biphotochromic nature can be observed on the wild type gene, while other biphotochromic proteins (IrisFP^22^, NijiFP^23^, etc.) were obtained by site-directed mutagenesis of amino acid residues in the chromophore microenvironment (M159A, F173S) in contrast to SAASoti, which does not require these mutations and nevertheless has a biphotochromic nature. This fact makes SAASoti a unique representative of GFP-family. A monomeric variant of SAASoti (mSAASoti) was also obtained by site-directed mutagenesis (V127T) and successfully applied as a genetically encoded fluorescent marker in PALM-technique^24^. SAASoti crystal structure has not been obtained yet, but we constructed a 3D model (Fig. 1) based on the known structure of another biphotochromic protein IrisFP (PDB ID: 2VVH)^22^. According to the data, presented in supplementary information of^24^, formed at high concentrations, weak dimers of V127T SAASoti can be disrupted by the addition of reducing agent dithiothreitol (DTT, 10 mM) to the sample.

According to the structure on Fig. 1 five cysteine residues are located in the SAASoti structure—C21, C71, C105, C117, and C175. Structure analysis of the SAASoti 3D-model revealed three surface-facing cysteine residues (C21, C71, and C117), two of them—21 and 117—might be involved in the dimer formation process, as they are located in the dimer interface. Sequence alignment (Table 1) with other homologous fluorescent proteins—moxDendra2, moxEos3.2, moxMaple3—revealed that SAASoti is the only FP possessing cysteine residue in the position 21 (SAASoti numbering), whereas in other proteins asparagine occupies the corresponding position. Amino acid residues serine or threonine occupy 117 position in other cases. For this reason, we generated C21N and C117S SAASoti variants by site-directed mutagenesis. After protein expression and purification steps we performed size-exclusion chromatography of the mutant variants in order to analyze their aggregation state. As it can be seen from the chromatogram on Figure S2, the resulted protein—C21N mSAASoti—even at higher concentrations (0.35 mM vs 0.22 mM for mSAASoti) exists as a monomer. Thus, C21N substitution was shown to disrupt weak dimers at higher concentrations of the protein.

**Table 1:**
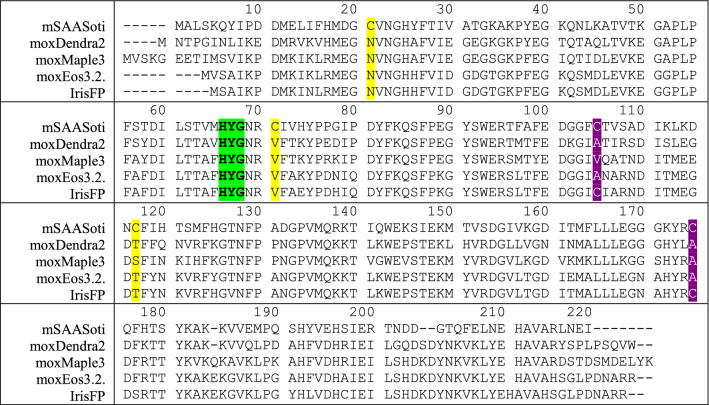
Sequence alignment of different FPs.

The cysteine residues mutated in this study are colored in yellow if the side chain is oriented to the solution and in magenta if the side chain is inside the β-barrel, chromophore is highlighted in green. Sequence alignment was performed using https://www.ebi.ac.uk/Tools/msa/clustalo/ algorithm.

As there are several cysteine residues in the SAASoti structure, we tested the effect of their individual, double and triple substitutions. Based on the previous data^25,26^, we obtained the following mSAASoti variants: C71V, C105V, C175A, C21N/C71V, C21N/C175A and C21N/C71V/C175A. The corresponding mutant forms were generated by site-directed mutagenesis; the recombinant proteins were expressed and purified as described below. From all the forms C21N/C71V/C175A mSAASoti turned out to lose its fluorescence. After applying site-saturation mutagenesis, we obtained C21N/C71G/C175A SAASoti mutant with greater brightness and faster photoconversion kinetics.

At first, we measured different physicochemical parameters [excitation/emission maxima λ_ex_/λ_em_, pK_a_ values of the chromophore, molar extinction coefficient (ε) and quantum yield (ϕ)] of the obtained SAASoti mutant variants. Introduced point mutations did not lead to the spectral shift of the green forms (λ_ex_/λ_em_ = 509/519 nm). As it can be seen from Table 2, C21N/C71V SAASoti has the minimum value of the molar extinction coefficient, quantum yield does not differ much when switching between the mutants.

**Table 2:** Physicochemical properties and fluorescent parameters of different mSAASoti mutant forms.

	pK_a_	ε, M^−1 ^* cm^−1^	ϕ	Brightness (ϕ * ε)
mSAASoti	6.3 ± 0.1	75.0	0.59 ± 0.02	44.3
C21N	6.4 ± 0.1	82.4	0.61 ± 0.02	50.3
C105V	6.5 ± 0.1	61.0	0.60 ± 0.02	36.6
C71V	6.5 ± 0.1	65.1	0.63 ± 0.04	41.0
C175A	6.7 ± 0.1	80.1	0.55 ± 0.05	44.0
C117S	6.2 ± 0.1	66.3	0.54 ± 0.03	35.8
C21N/C71V	6.3 ± 0.1	48.9	0.58 ± 0.02	28.4
C21N/C175A	6.3 ± 0.1	65.4	0.55 ± 0.03	36.0
C21N/C71G/C175A	6.4 ± 0.1	83.8	0.60 ± 0.02	50.3

**Figure 2:**
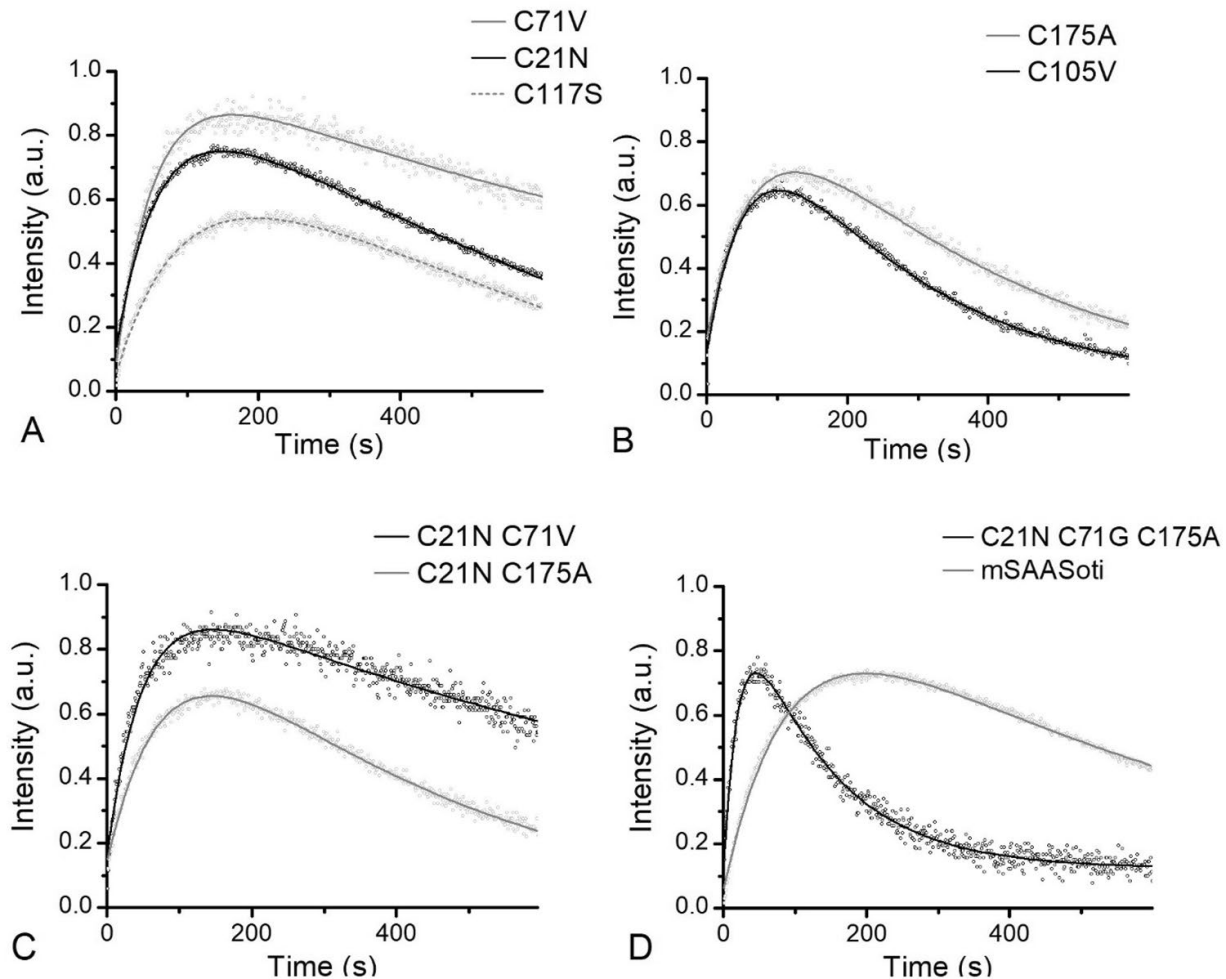
Green-to-red photoconversion kinetics of mSAASoti variants (λ_em_ = 590 nm) recorded during 10 min of 400 nm (146 mW/cm^2^) illumination. Dots—experimental data, line—fitting. Proteins with a single mutation of Cys side chains oriented: (**A**) to the solution; (**B**) inside the β-barrel; (**C**) double mutations; (**D**) triple mutation. Fluorescent intensity is normalized to pre-exponential value I according to Eq. (1).

Obtained mutant forms were tested to different types of phototransformations—irreversible green-to-red photoconversion and reversible photoswitching between fluorescent (on) and dark (off) states. Green-to-red photoconversion occurs under 400 nm illumination. We recorded emission spectra of the samples under 400 nm illumination during 10 min (Fig. 2) and analyzed red fluorescence signal (λ = 590 nm) from the time kinetic curves.

The process can be described with a bi-exponential model (Eq. 1). The derivation of Eq. (1) is presented in Supplementary Information),

1 $$I_{red} \left( t \right) = I*\frac{k1}{{k2 - k1}}*\left( {\exp \left( { - k1*t} \right) - \exp \left( { - k2*t} \right)} \right) + c$$

where the first exponent is responsible for the red form formation, while the second exponent describes its photodestruction, c—background and residual signal. The corresponding kinetic parameters for each mutant form are presented in Table 3. The maximum photoconversion rate is observed for all the forms containing cysteine substitution in the 71st position (C71V, C21N/C71V, and C21N/C71G/C175A), along with that in the case of single and double mutants C71V substitution also affected their photostability (minimum k_2_ value). C21N/C71G/C175A SAASoti has the maximum k_1_ and k_2_ values, indicating on both processes of photoconversion and photodestruction proceed faster. All the mutant forms except C117S variant converted to the red form faster or with the same rate. In the case of C105V substitution, it also negatively affected its photostability. Interestingly, SH-group of 117 cysteine residue is proposed to be surface-facing, but its substitution to serine affected photoconversion kinetics. The increase in the initial rate suggests the reduction of the 400 nm exposure time, that will be less toxic to live cells.

**Table 3:** Kinetic parameters for the on-to-off photoswitching reaction, off-to-on recovery and green-to-red photoconversion calculated for different SAASoti mutant forms.

*SAASoti form*	On-to-off photoswitching						Off-to-on recovery^c^	Green-to-red photoconversion^d^	
	1st cycle^a^			2nd cycle^b^					
	I_2_/I_1_	k_1 _* 10^3^, s^−1^	k_2 _* 10^3^, s^−1^	I_2_/I_1_	k_1 _* 10^3^, s^−1^	k_2 _* 10^3^, s^−1^	k * 10^3^, s^−1^	k_1 _* 10^3^, s^−1^	k_2_^*^10^3^, s^−1^
mSAASoti	0.27	5.5 ± 0.2	9.8 ± 0.7	0.26	7.8 ± 0.1	24.7 ± 0.8	22 ± 1	11 ± 0.1	2 ± 0.01
C21N	0.27	5.7 ± 0.1	15.7 ± 0.1	0.76	5.3 ± 0.1	13.7 ± 0.2	9.5 ± 0.2	14 ± 0.1	2 ± 0.02
C21N/C71V	0.26	6.8 ± 0.1	15.6 ± 0.6	0.2	7.4 ± 0.1	22.8 ± 0.8	12.5 ± 0.2	22 ± 0.4	1 ± 0.02
C71V	0.14	4.5 ± 0.2	14.2 ± .2.0	0.4	5.1 ± 0.2	15 ± 2	15.5 ± 0.2	21 ± 0.3	1 ± 0.02
C105V	0.22	9.1 ± 0.1	34.3 ± 0.7	2.06	7.1 ± 0.2	17.9 ± 0.2	17.9 ± 0.4	15 ± 0.4	5 ± 0.01
C117S	0.09	6.4 ± 0.1	10.9 ± 0.3	0.24	6.4 ± 0.1	15.6 ± 0.5	9.7 ± 0.3	9 ± 0.1	3 ± 0.05

	1st cycle^b^			2nd cycle^b^					
C175A		3.5 ± 0.1		0.12	3.1 ± 0.1	16 ± 1	5.5 ± 1.2	15 ± 0.2	3 ± 0.03
C21N/C175A	–	4.1 ± 0.1	–	0.98	1.5 ± 0.3	7.5 ± 0.3	2.5 ± 0.1	13 ± 0.2	3 ± 0.03
C21N/C71G/C175A	–	4.0 ± 0.1	–	–	4.4 ± 0.1	–	4.5 ± 0.1	46 ± 2	9 ± 0.1

^a^Photoswitching kinetics is described by Eq. (2).

^b^Photoswitching kinetics is described by Eq. (3).

^c^Thermal relaxation recovery as absorption at 509 nm is described by Eq. (4).

^d^Photoconversion kinetics is described by Eq. (1).

It is well known that photoswitching between the fluorescent and dark states occurs under 470 nm light (in the case of the green form) illumination and is associated with cis–trans isomerization of the chromophore, accompanied with the change in its protonation state^27^. The resulting conformational state is thermodynamically less stable, and switched-‘off’ protein undergoes either thermal relaxation (τ_1/2_ = several hours) or can be switched-‘on’ by 400 nm light (within seconds). We exposed solutions of the obtained mutant forms to 470 nm light in cuvette and recorded the decrease in the green (519 nm) fluorescence intensity over the illumination time.

As it can be seen from the photoswitching kinetics presented on Fig. S3, C105V mSAASoti demonstrates the maximum photoswitching rate, whereas mSAASoti variants with C175A substitution (single, double, and triple mutants)—the minimum. It is important to note, that the same sample can be repeatedly photoswitched between fluorescent ‘on’ and dark ‘off’ states for several times. As the mechanism of green-to-red photoconversion involves protonated form of the chromophore, and in more acidic solutions photoconversion proceeds faster, protein samples were prepared in 20 mM NaHCO_3_ (pH 9.2) in order to minimize red photoconversion efficiency during green fluorescence regeneration by 400 nm light. The samples were sequentially illuminated with 470 nm light (10 min) and 400 nm light (10 s). Figures S3 and S5 show that photoswitching kinetics of the on-to-off cycle are identical from cycle to cycle for C175A-containing mSAASoti forms, whereas in all the other cases we observed acceleration of the photoswitching rate when going from the first switching cycle to the second.

**Figure 3:**
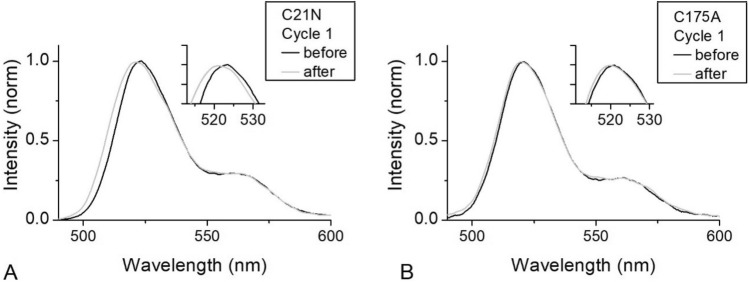
Emission spectra of (**A**) C21N mSAASoti and (**B**) C175A mSAASoti before (black line) and after (grey line) 500 s of 470 nm illumination.

Kinetic curves of the first photoswitching cycles can be described by a bi-exponential model (Eq. 2).

2 $$I = I_{1} {*}\exp \left( { - k_{1} t} \right) - I_{2} {*}\exp \left( { - k_{2} t} \right) + c$$

where the first component describes switching and the second component is responsible for green fluorescence intensity increase at the fixed wavelength 519 nm (Fig. 3). When comparing emission spectra of the mutant forms before and after 470 nm exposure (500 s) we observed a noticeable spectral blue-shift, which is amplified in subsequent switching cycles (Fig. S4, Table S2, Table S3). The first photoswitching cycle has a higher spectrum shift which may be the main reason for the negative exponent. It is noteworthy, that this phenomenon was not observed for all C175A-containing mSAASoti forms, which allows us to assume the photooxidation of the corresponding cysteine residue.

Starting from the second photoswitching cycle a bi-exponential model (Eq. 3) describes the process.

3 $$I = I_{1} {*}\exp \left( { - k_{1} t} \right) + I_{2} {*}\exp \left( { - k_{2} t} \right) + c$$

It is noteworthy, that all mSAASoti variants with C175A substitution do not have this increase in green fluorescence emission in the first on-to-off cycle and do not have any spectral changes (Fig. 3B). The second photoswitching kinetics of C175A and C21N/C175A mSAASoti is described by a bi-exponential model (Eq. 3), but in the case of triple mSAASoti variant with C175A substitution kinetics does not have the second component at all (Table 3, Figure S3).

As it can be seen from Figure S5, all SAASoti variants containing C175A substitution lose their initial fluorescence value when switching to subsequent photoswitching cycles, indicating on some photodestruction process. In other words, substitution of the 175 cysteine residue results in the photostability loss. Moreover, all these forms can be photoswitched to a lower degree in comparison with the other forms and mSAASoti.

We studied thermal relaxation of all obtained mSAASoti forms by absorption spectra registration. In the case of mSAASoti it takes hours (τ_1/2_ = 50 min at 22 °C)^20^. Relaxation kinetics for all other forms are presented as A_509_ maximum (anionic form) recovery in time (Fig. S6). Relaxation kinetics can be described by a monoexponential model (Eq. 4):

4 $$A = A_{0} *\left( {1 - \exp \left( { - k*t} \right)} \right) + c$$

Table 3 summarizes the rate value constants calculated for different mutant forms. The C105V substitution had minimal effect on the relaxation rate. In the case of C21N and C117S substitutions thermal relaxation proceeds more slowly, and the variants with the C175A substitution have the lowest thermal relaxation rate.

### Molecular dynamics simulations of the C175A variants of mSAASoti

**Figure 4:**
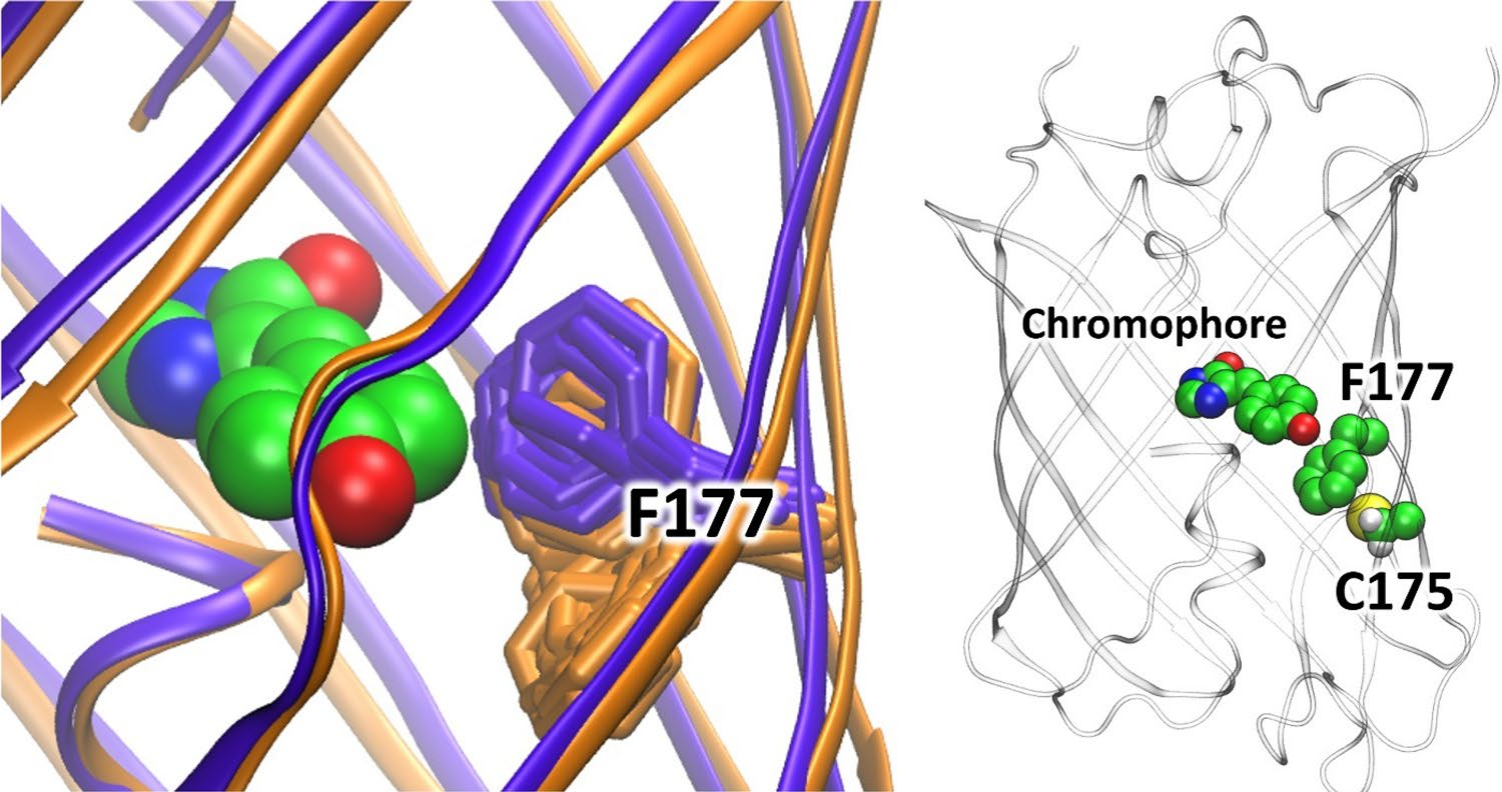
Left panel: Alignment of molecular models of the mSAASoti (orange) and C175A variant of SAASoti (violet). Conformations of F177 along MD trajectories are shown in stick representation. Right panel: The 3D structure of the entire mSAASoti: the chromophore, F177 and C175 are highlighted (right panel).

According to the experimental studies a single C175A mutation results in the most pronounced decrease of the relaxation rate of the green form from the dark to the fluorescent state (Fig. S6, Table 3). To understand the origin, we performed classical molecular dynamic simulations of the mSAASoti and C175A variants of mSAASoti, 200 ns each. We compare the dynamic behavior of both of these systems by analysis of RMSD and flexibility of amino acid residues in the chromophore region (Fig. 4). We performed alignment of all heavy atoms from all MD frames for both proteins. The standard deviation of the RMSD calculated for only backbone atoms is smaller in the WT protein, indicating that the protein fold is more rigid in it. Contrary, the RMSD values calculated for the side chain atoms are characterized by larger standard deviation of 0.25 Å in the wild type compared with the 0.18 Å in the C175A mutant. The latter is an indication that changes of the side chain flexibilities might be responsible for the different rates of the fluorescent state recovery in the green form. We analyzed dynamics of the side chains of all residues and found that the F177 behaved differently in these two proteins (Fig. 4). It is located between the phenyl part of the chromophore and the 175th residue (Fig. 4). Close contacts between the 175th residue and F177 clarify the origin of the observed influence of C175A mutation on the conformational dynamics of F177. Its side chain in the mSAASoti species demonstrates a wide range of conformations that is also revealed by the large value of the standard deviation of RMSD, 1.49 Å. In C175A the F177 side chain is less flexible and exhibits a set of similar conformations with the standard deviation of RMSD being threefold smaller. In the C175A variant the major conformation of the F177 side chain shapes a tighter binding pocket in the chromophore phenyl fragment region that should hinder the thermal relaxation. Contrary, for the mSAASoti, the F177 has a high population of the side chain conformations that are farer from the chromophore that simplify the isomerization process.

### The origin of the pK_a_ change in the red form of C21N mutant

**Figure 5:**
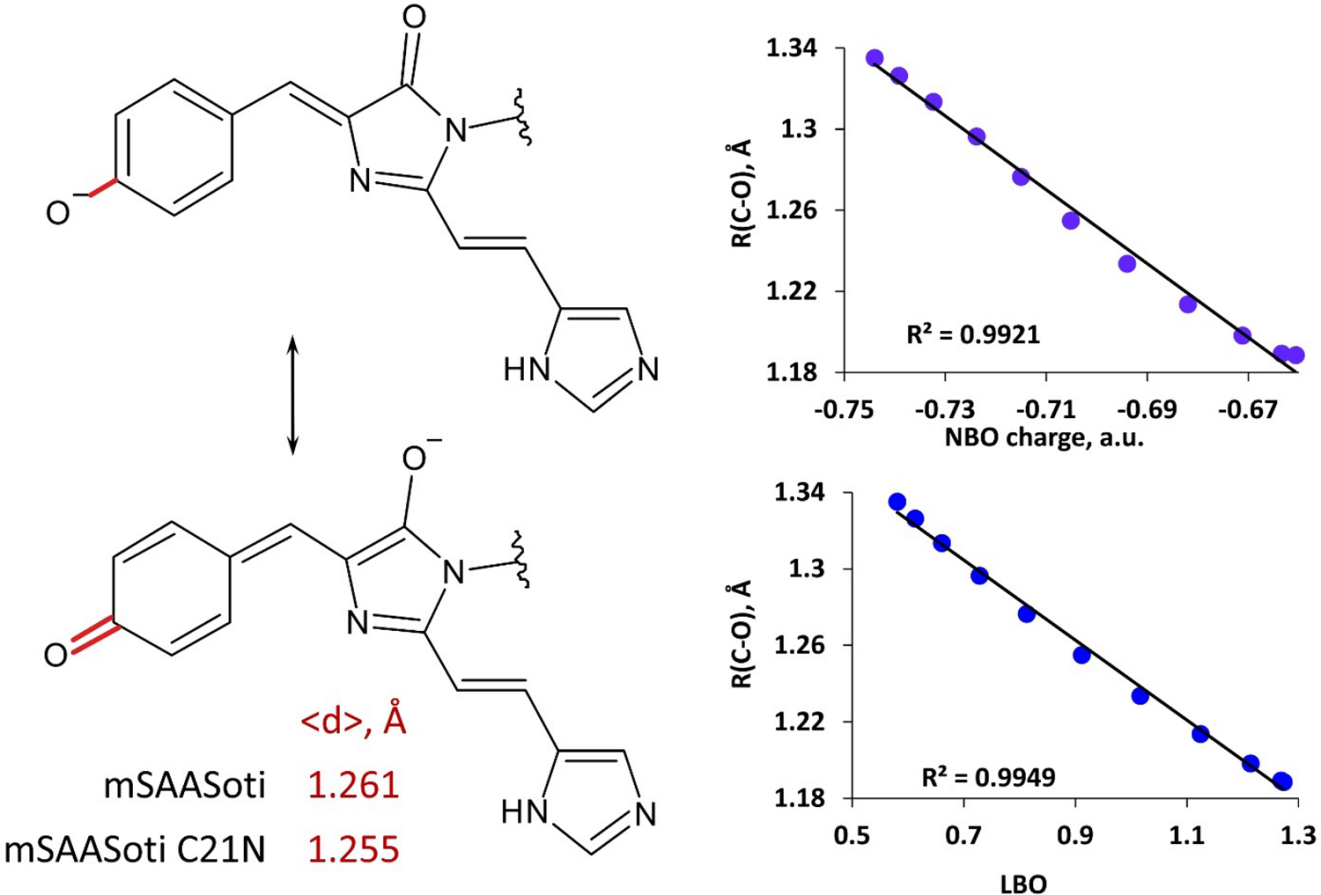
Two resonance forms of the anionic chromophore (left). The C–O bond of the phenyl fragment is shown in red. Its distance is longer (single bond) if the negative charge is located on the phenyl oxygen and shorter (double bond) if the negative charge is located on the imidazolinone oxygen. The mean distances, < d > , along the QM/MM MD trajectories are shown in the inset. Right panels demonstrate correlations between the electron density-based parameters, NBO charge (top) and LBO (Laplacian bond order, bottom), and the C–O bond distance.

The C21 residue is located far from the chromophore (Fig. 1). Moreover, its side chain is oriented to the solution and experimentally observed kinetic behavior of C21N mutant is similar to that of the mSAASoti. However, the pK_a_ of the red form changes from 6.6 to 7.5 upon introducing this mutation (Table S4). We performed molecular dynamics simulations with the QM/MM potentials of the red forms of both of these proteins to find the quantities that are responsible for the variations of the pK_a_ values. The anionic form of the chromophore may exist in various resonance states (Fig. 5). The pK_a_ of the chromophore should decrease if the equilibrium is shifted to the tautomer with the negative charge being localized on the phenolate oxygen (Fig. 5). We analyzed dynamic behavior of the chromophore in both mutants and studied the correlations between the geometry parameter of the R(C–O) covalent bond distance, the bond order calculated from the Laplacian of electron density (LBO) and NBO charge. The mean C-O distance in the phenyl fragment of the chromophore is 1.261 Å in the mSAASoti protein and 1.255 Å in the C21N mutant (Fig. 5). We extracted several frames from the MD trajectory and evaluated electron density-based quantities that might be responsible for the pK_a_ variations. Figure 5 demonstrates correlation between the NBO charge and R(C–O). The increase of the covalent bond distance results in the shift of the NBO charge to the more negative values, i.e., the predominance of the resonance form with the phenolate anion. Another criterion, the Laplacian bond order accounts for various bonding feature including bond polarity. It is evaluated by integration of the negative part of the Laplacian of electron density multiplied by weighting functions proposed by Becke, taken with the opposite sign. The more negative is the Laplacian of electron density in the covalent bond region, the more is electron density concentration and the stronger is covalent bond. It corresponds to the larger values of the of LBO. Also, the decrease of LBO is the measure of the increase of the bond polarity. Therefore, the latter increases with the increase of the C-O distance, that is in line with other computational results. Thus, the studies of the local properties of the chromophore assist in the understanding of the pK_a_ variations even caused by the distant amino acid mutations.

## Discussion

Characterization of physicochemical and fluorescent parameters (λex/λem, pK_a_, ε, φ) demonstrated that incorporated amino acid substitutions, even of the surface-facing cysteine residues, greatly affected different mSAASoti properties. E.g., C21N and C175A substitutions led to a significant shift of the pKa value of the red form to more alkaline pH values. By applying molecular dynamics simulations with the QM/MM potentials of the red forms of C21N and mSAASoti variants we estimated that higher pK_a_ value of the red form in the case of C21N SAASoti is associated with the mean C-O length shortening in the phenyl fragment of the chromophore. As for surface cysteine residues (C21 and C117) it was found that C21 residue is responsible for mSAASoti partial dimerization at high concentrations, its C21N substitution to mSAASoti gene resulted in the stabilized monomeric form. That is why C21N was chosen as a new improved mSAASoti variant. Forms with substituted 71 cysteine demonstrated the increased rate of green-to-red photoconversion, and triple mSAASoti mutant—the maximum rate, but under 400 nm illumination the photostability of the generated red form is greatly reduced in this case. Green-to-red photoconversion is a photochemical reaction, resulting in the chromophore transformation from a two-ring π-conjugated electron system into a three-ring one. Firstly, C71 is located in the close proximity to the chromophore compared to other cysteine residues, moreover, it follows highly conservative for photoconvertible FPs arginine residue 66 (numbering according to EosFP, R70 in the case of SAASoti, Table 1). It is also known from the literature, that R66 may play a role in the transient dark state formation^28^ and photobleaching^23,29,30^. Microenvironment of the chromophore (H193, E211) serves as the catalyst in this photochemical reaction^31^. C71V substitution can affect the stereometry of the bonds around the chromophore. C71V substitution might cause some alteration of the neighboring residues which in its turn can affect cooperation between the residues in highly conservative tetrad R66-E144-H193-E211 and the reaction rate as well. An interesting observation is that C117S mSAASoti converts to the red form with the slowest rate, whereas this amino acid is a ‘surface-facing’ one. Some allosteric effect might take place in this case. The authors^25^ reported the minor increase in the rate and extent of photoconversion for moxDendra2 in comparison with Dendra2 and the phenomenon was explained by improvement in extinction coefficients for both green and red forms of moxDendra2. According to SAASoti 3D-model C105 side chain is buried inside β-barrel and this amino acid seems to be far from the chromophore, nevertheless C105V variant demonstrated the fastest photoswitching and relaxation kinetics, perhaps in this case some allosteric effect takes place. All mSAASoti variants containing C175A substitution demonstrated different photoswitching kinetics, and we did not observe any spectral shift when going to the subsequent photoswitching cycles, indicating on some possible photochemical modification (oxidation) of 175 cysteine residue in the rest mutant forms. C175A is a substitution in the vicinity of chromophore, the authors^29^ showed that not only M159 could be oxidized under blue light illumination, but also the corresponding cysteine residue (171 in the case of IrisFP). Bi-exponential nature of ‘on-to-off’ switching was explained earlier^32^ by existing of two protein populations both having individual switching kinetics. Double and triple mutants containing C175A substitution both follow a mono-exponential law, whereas photoswitching kinetics of monosubstituted C175A SAASoti is described by a bi-exponential function with both components having the same sign. Along with the fact that forms with C175A substitution do not have any spectral shift, as we can assume, cysteine 175 can be photo-oxidized during the first photoswitching cycle and then this new population also undergoes photoswitching. The probable reaction scheme and the corresponding kinetic equation are presented in Supplementary Information. Thermal relaxation of C175A mSAASoti variants also proceeds more slowly in comparison with mSAASoti. Interestingly, the authors^29^ demonstrated that depending on light power intensity two different mechanism of photobleaching can be realized. E.g., illumination at high laser-light intensity (∼ 0.1 kW/cm^2^, 488 nm) resulted in the glutamate 212 decarboxylation and significant permutation of the H-bonding around the chromophore and also its notable modification, whereas under low-intensity illumination (∼ 10 W/cm^2^, 488 nm) the authors observed oxidation of sulfur-containing residues M159 and C171, locking the chromophore in the dark state. However, C175A substitution negatively affected mSAASoti’s photostability during repeated photoswitching cycles. C175 is located between and slightly above the residues M163 and F177 (M159 and F173, EosFP), which were substituted to alanine and serine, respectively, in order to obtain photoswitchable variants of the photoconvertible proteins. We assume that C175A substitution might lead to steric constraints complicating the photoisomerization reaction.

Classical molecular dynamic simulations of the mSAASoti and C175A variant with subsequent analysis of the cross-correlation maps, RMSD and flexibility of amino acid residues in the chromophore region was performed. Amino acid residues in the mSAASoti protein demonstrated more correlated motions in comparison with the C175A variant. It was also established that F177 side chain in the case of C175A mSAASoti is less flexible, moreover this phenylalanine mainly occupies the same spatial position in C175A as the phenyl part of the chromophore does in the dark state. In other words, the photoswitching reaction in the mSAASoti is facilitated by F177 conformational flexibility.

Taking into account all data obtained we can assume that C71 residue is sensitive to green-to-red photoconversion as its substitution accelerates the reaction, while C105 and C175 amino acid residues deal with reversible photoswitching, and their replacement increase or decrease the reaction, respectively. The rate constants corresponding to the irreversible green-to-red photoconversion and thermal relaxation from dark to fluorescent state differ up to one order of magnitude between mSAASoti variants. Sometimes even substitutions of amino acid residues located at a significant distance from the chromophore affects the nature of phototransformation (C117S). Kinetic analysis of the phototransformation reactions (photoconversion and photoswitching) contributed a lot to detect differences in mechanisms and between different mutant forms. To conclude, we demonstrated the influence of the allosteric regulation on photophysical and photochemical properties of biphotochromic protein mSAASoti.

## Materials and methods

### Site-directed and site-saturated mutagenesis

mSAASoti mutant forms C21N, C117S, C117T, C71V, C105V, C175A, C21N/C71V, C21N/C175A, C21N/C71V/C175A were generated by overlap extension PCR using designed primers and C21N/C71G/C175A was generated by site-saturated mutagenesis using degenerate primers. The corresponding genes were cloned into pET22b vector for protein expression in bacteria and further purified as described earlier^24^.

### Protein expression and purification

All SAASoti mutant forms were expressed in *E. coli* BL21 (DE3) cells as previously described^26^. Cells were harvested by centrifugation (4000 g, 15 min, 4° C) and resuspended in 20 mM Tris–HCl, 150 mM NaCl pH 7.4. After the cells were disrupted using a French-press (Thermo Scientific), the lysate was centrifuged for 15 min, (30,000 g, 4 °C). The proteins were precipitated by salting out the colored supernatant by adding ammonium sulfate to 50% saturation, and incubated overnight at 4 °C. A colored precipitate was collected by centrifugation at 3000 g, resuspended in 20 mM Tris–HCl, 1 M (NH_4_)_2_SO_4_ to completely clear solution. Protein solutions were loaded onto HiPrep Butyl FF 16/10 column in 20 mM Tris–HCl, 1 M (NH_4_)_2_SO_4_ (buffer A), proteins were eluted using a decreasing salt gradient (20 mM Tris-HC, buffer B). All collected fractions containing SAASoti variants were desalted and concentrated by using Amicon Ultra-15 10 kDa columns (Merck, Millipore) in 20 mM NaHCO_3_ (buffer C). The next purification step included anion-exchange chromatography on MonoQ 5/50 GL column, SAASoti was eluted with a NaCl gradient (20 mM NaHCO_3_, 0.5 M NaCl, buffer D). All chromatographic steps were performed on AKTAPurifier 10 system (GE Healthcare). Thanks to this purification scheme, we obtained protein fractions of different SAASoti mutant forms with high purity (A_280_/A_509_ > 1/3). Protein purity was also confirmed by gel-electrophoresis. SDS-PAGE was conducted in a Bio-Rad Mini Protean II system using hand-poured gradient (4–15%) polyacrylamide gels in a standard Tris-Gly buffer containing 0.1% SDS. Molecular weights were determined by using SM0431 Fermentas unstained protein markers. Gels were stained with PageBlue Thermo Scientific reagent.

*Absorbance spectra* were registered using a Cary 60 (Agilent, USA) spectrophotometer at a constant temperature (22 °C) and a 3 mm quartz cuvette (Hellma, Germany), samples were in 20 mMTris–HCl, 150 mM NaCl, pH 7.4 (if not otherwise stated).

*Fluorescence spectra* were registered using a Cary Eclipse fluorescence spectrophotometer at the room temperature and a 3 mm quartz cuvette (Hellma, Germany) samples were in 20 mM Tris–HCl, 150 mM NaCl, pH 7.4 (if not otherwise stated).

*Green-to-red photoconversion and reversible photoswitching kinetic experiments* were carried out using a self-made setup constructed with Lumencor Spectra X LED light source, λ = 400 nm (146 mW/cm^2^) for green-to-red photoconversion with adding 550 nm (20 mW/cm^2^) light in some cases in order to increase signal/noise ratio (Supplementary Material, Fig. S1) and λ = 470 nm (167 mW/cm^2^) for *on-to-off photoswitching*, emission spectra were recorded by a homebuilt spectrometer Spectrum-Cluster based on linear CCD sensor. Protein solutions (10 μM) in 20 mM Tris–HCl (pH 7.4), 150 mM NaCl were placed in a thermostatically controlled (25° C) microcuvette (Hellma, Germany) with a 3 × 3 mm optical path and illuminated for 10 min with 400 or 470 nm light for conversion or switching experiments, respectively. Fluorescent intensity is normalized to I max value (Imax = I * (k_1 _− k_2_)/k_1_). The normalization was performed using the model of a monomolecular consecutive process of the red form formation and destruction, 1 reflects max fluorescence intensity of the red form if there were no photodestruction. (For more details see^24^).

*Oligomeric state determination of SAASoti mutant forms* was performed as described earlier^24^ using gel-filtration.

*Absorbance spectra* were registered with a Cary 60 (Agilent, USA) spectrophotometer at a constant temperature (22 °C) and a 3 mm quartz cuvette (Hellma, Germany), samples were in 20 mM Tris–HCl, 150 mM NaCl, pH 7.4 (if not otherwise stated).

*Thermal relaxation* was studied by recording absorption spectra for thermal relaxation on a Cary 300 Bio spectrophotometer (Varian). Data analysis was performed with Origin 8.5 software package.

### Photoswitching cycles

Switching cycles were performed by illumination the protein solution in microcuvette (Hellma, Germany) with optical path 3 × 3 mm by λ = 470 nm (167 mW/cm^2^) light for 10 min in the case of ‘on-to-off’ photoswitching with subsequent illumination at 400 nm (21.3 mW/cm^2^) light for 10 s for regeneration of the fluorescent form. Protein samples were prepared in 20 mM NaHCO_3_ pH 9.2 in order to minimize the amount of the protonated form and to avoid photoconversion under 400 nm illumination. Light source and fluorescence detector were the same as in “*Green-to-red photoconversion and reversible photoswitching kinetic experiments”.* Fluorescence intensity is normalized to Imax value (I_norm_ = I/I_max_).

### Classical MD simulations

The 3D structures of the WT, C21N and C175A variants of SAASoti for the MD trajectory were constructed from the available crystal structure of the green cis form of the IrisFP (PDB ID: 2VVH)^22^. The sequence alignment and amino acid replacements were performed using MODELLER^33^ implemented in Chimera^34,35^. The protonation states of amino acid residues were assigned using PROPKA software^36^. The CHARMM36^37,38^ force field parameters were utilized for protein and the CGenFF^39^ force field parameters for the chromophore in the green form. The system was solvated in the rectangular water box with the TIP3P^40^ water molecules and neutralized by adding sodium ions. Classical molecular dynamics simulations were performed in the NAMD 2.13 software package^41^. Each system was preliminary equilibrated by 10,000 minimization steps and 20 ns MD run. Production runs for the WT and C21N variants of SAASoti were performed for 200 ns with 1 fs time step in the NPT ensemble at p = 1 atm and T = 300 K. The pressure and temperature were controlled by Nosé-Hoover barostat and Langevin thermostat, respectively. To decrease the influence of error accumulation for such long trajectories, we randomly reassigned velocities every 40 ns. The cutoff distances were 12 Å for both electrostatic and van der Waals interactions with switching to the smoothing function at 10 Å.

### MD simulations with QM/MM potentials

The systems for the QM/MM (combined quantum mechanics/molecular mechanics) MD simulations were preliminary equilibrated in classical MD runs as described above. The MM subsystems were described similarly to the classical MD. The QM part was composed of the chromophore, the side chains of Gln42, The63, Arg70, Arg95, Ser146, His197, Glu213 (in the neutral form) and two water molecules. The green to red conversion was manually performed and these coordinates were used as initial for the QM/MM MD runs. The system was preliminary minimized for 100 steps. After that the 5 ps production runs were performed. The QM part was described at the ωB97X-D3/6-31G** Kohn–Sham DFT level^42^. The QM/MM MD simulations were performed using the interface^43^ for the classical MD software NAMD^41^ and a quantum chemistry package TeraChem^44^. The NBO^45^ analysis at MD frames was performed within the TeraChem^44^. The Laplacian bond orders^46^ were calculated using the Multiwfn program^47^.

## Supplementary Information

Supplementary Information.
