# Supplementary material for: The role of cysteine residues in the allosteric modulation of the chromophore phototransformations of biphotochromic fluorescent protein SAASoti
Source: Sci Rep. 2021 Dec 21;11:24314. doi: 10.1038/s41598-021-03634-9 (PMC8692419; doi:10.1038/s41598-021-03634-9)
Supplement: Supplementary file 1 — Supplementary Information. [file 41598_2021_3634_MOESM1_ESM.docx]

**The Role of Cysteine Residues in the Allosteric Modulation of the Chromophore Phototransformations of Biphotochromic Fluorescent Protein SAASoti**

**Gavshina A.V.^1^, Marynich N.K.^1^, Khrenova M.G.^1,2^, Solovyev I.D.^1^, Savitsky A.P.^1, 2^ ***

^1^A.N. Bach Institute of Biochemistry, Research Center of Biotechnology of the Russian Academy of Sciences, Moscow, Russia

^2^Lomonosov Moscow State University, Department of Chemistry, Moscow, Russia

**Supplementary data**

***The derivation of equation*** 1:

In our experiment the red form was obtained by continuous illumination of protein solutions in a cuvette at 400 nm light (146 mW/cm^2^) with adding 550 nm (20 mW/cm^2^) light in some cases in order to increase signal/noise ratio. Red form fluorescence can be excited by both light bands with different efficiency. In our previous work [unique 2019] it was demonstrated, that only green form of mSAASoti can be photoswitched to the dark state under 470 nm light, whereas during 550 nm light illumination the red form irreversibly destructed without switching.

We also compared normalized kinetics received with or without additional green light. There is no visible difference between (Fig. 1). Thus, we suppose that the red form switching is negligible in our experiment. We cannot consider all the processes and use minimal kinetic scheme with apparent constants.


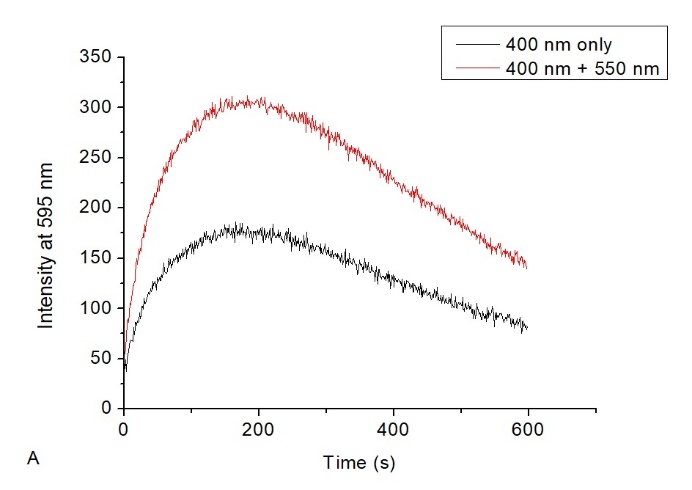

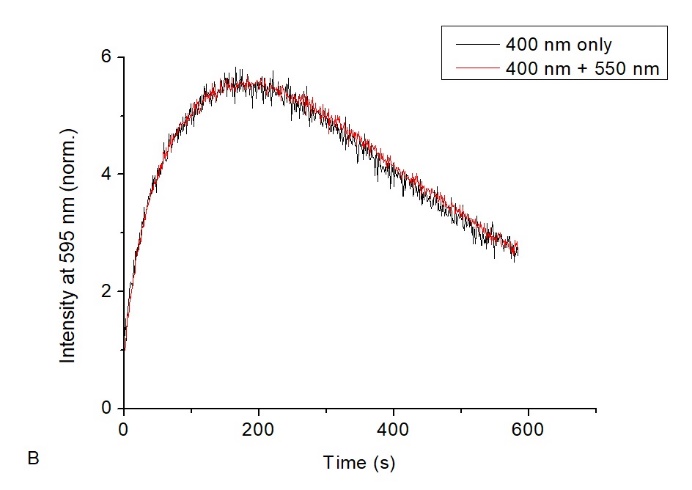


**Fig. S1.** Photoconversion kinetics of C117S mSAASoti at 400 nm with and without additional 550 nm illumination. A) 595 nm intensity curves, B) normalized curves comparing

Kinetic scheme: $A\underset{\to}{k1}B\underset{\to}{k2}C$, where A – green form, B – red form, C – destructed form, k1 – photoconversion under 400 nm illumination, k2 – photodistruction under 400 nm illumination. It is a pseudo unimolecular consecutive reaction (light power density is constant).

According to the chemical reaction rate law:

$$\frac{dA}{dt}=-k1*\left[ A \right]$$

$$\frac{dB}{dt}=k1*\left[ A \right]-k2*\left[ B \right]$$

The first equation has a simple solution as separable:

$$A(t)=A_{0}\exp\left( -k1*t \right)$$

Make the substitution in the B rate equation:

$$\frac{dB}{dt}=k1*A_{0}*\exp\left( -k1*t \right)-k2*\left[ B \right]$$

It is a first order differential equation. Let B is multiplication *u*v* and make the substitution.

$$u^{'}*v+u*v^{'}+k2*u*v=k1*A_{0}*\exp\left( -k1*t \right)$$

$$u^{'}*v+u*\left( v^{'}+k2*v \right)=k1*A_{0}*\exp\left( -k1*t \right)$$

Let $v^{'}+k2*v=0$:

$$\frac{dv}{dt}=-k2*v$$

The result of integration from 0 to *t* (*v*(0)=0):

$$v=\exp\left( -k2*t \right)$$

Substitute it:

$$\frac{du}{dt}*\exp\left( -k2*t \right)=k1*A_{0}*\exp\left( -k1*t \right)$$

$$\frac{du}{dt}=k1*A_{0}*\exp\left( \left( k2-k1 \right)*t \right)$$

Integrate from 0 to *t*:

$$u=\int_{0}^{t} k1*A_{0}*\exp\left( \left( k2-k1 \right)*t \right)dt=A_{0}*\frac{k1}{k2-k1}*\left( \exp\left( \left( k2-k1 \right)*t \right)-1 \right)$$

The result B(t) equation:

$$B\left( t \right)=u*v=A_{0}*\frac{k1}{k2-k1}*\left( \exp\left( \left( k2-k1 \right)*t \right)-1 \right)*\exp\left( -k2*t \right)=A_{0}*\frac{k1}{k2-k1}*\left( \exp\left( -k1*t \right)-\exp\left( -k2*t \right) \right)$$

Fluorescence intensity of the SAASoti red form has a linear dependency from its concentration in low optical density solution:

$$I_{B}\left( t \right)=i_{B}*B\left( t \right)=i_{B}*A_{0}*\frac{k1}{k2-k1}*\left( \exp\left( -k1*t \right)-\exp\left( -k2*t \right) \right)$$

Where $i_{B}*A_{0}$ – max intensity of the red form reached without photodestruction (k2=0). We can obtain this parameter from pre-exponential factor (X) of data fitting by $X/\frac{k1}{k2-k1}$ equation and normalize intensity curves.


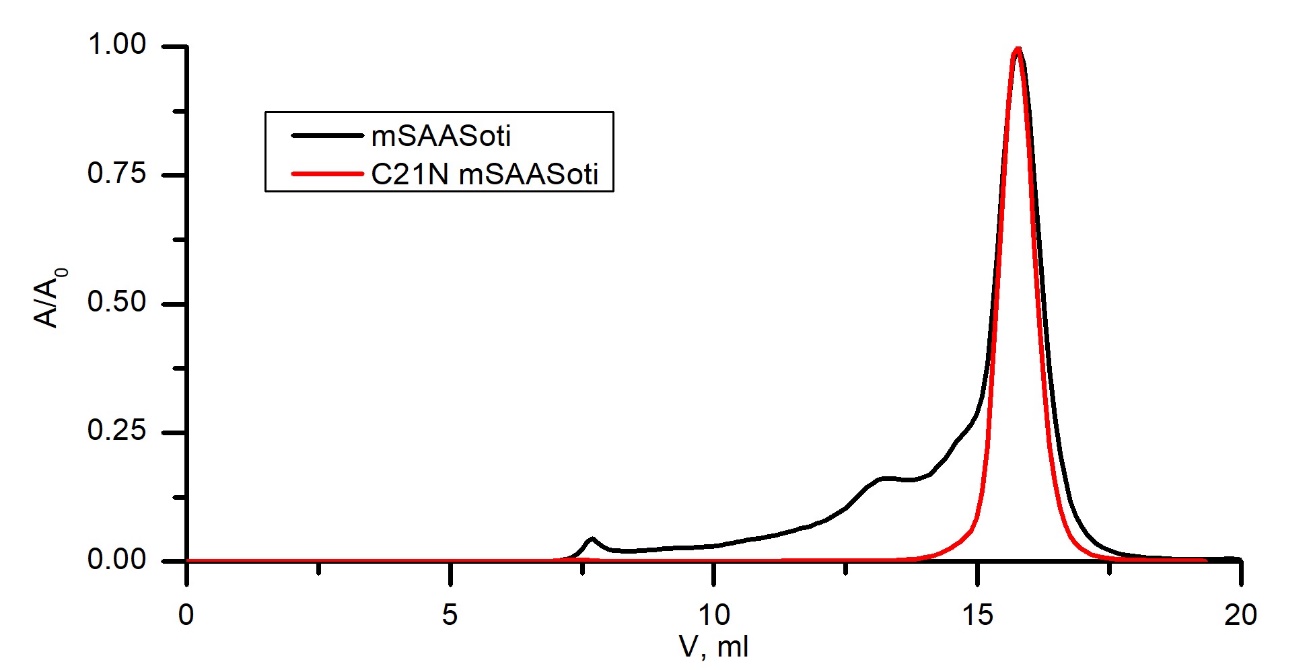


**Figure S2.** Size-exclusion chromatography of 0.22 mM mSAASoti (black line) and 0.35 mM C21N mSAASoti (red line). Superdex 200 100/20 GL column, detection by absorption at 509 nm, 20 mM Tris-HCl, 150 mM NaCl buffer (pH 7.4).


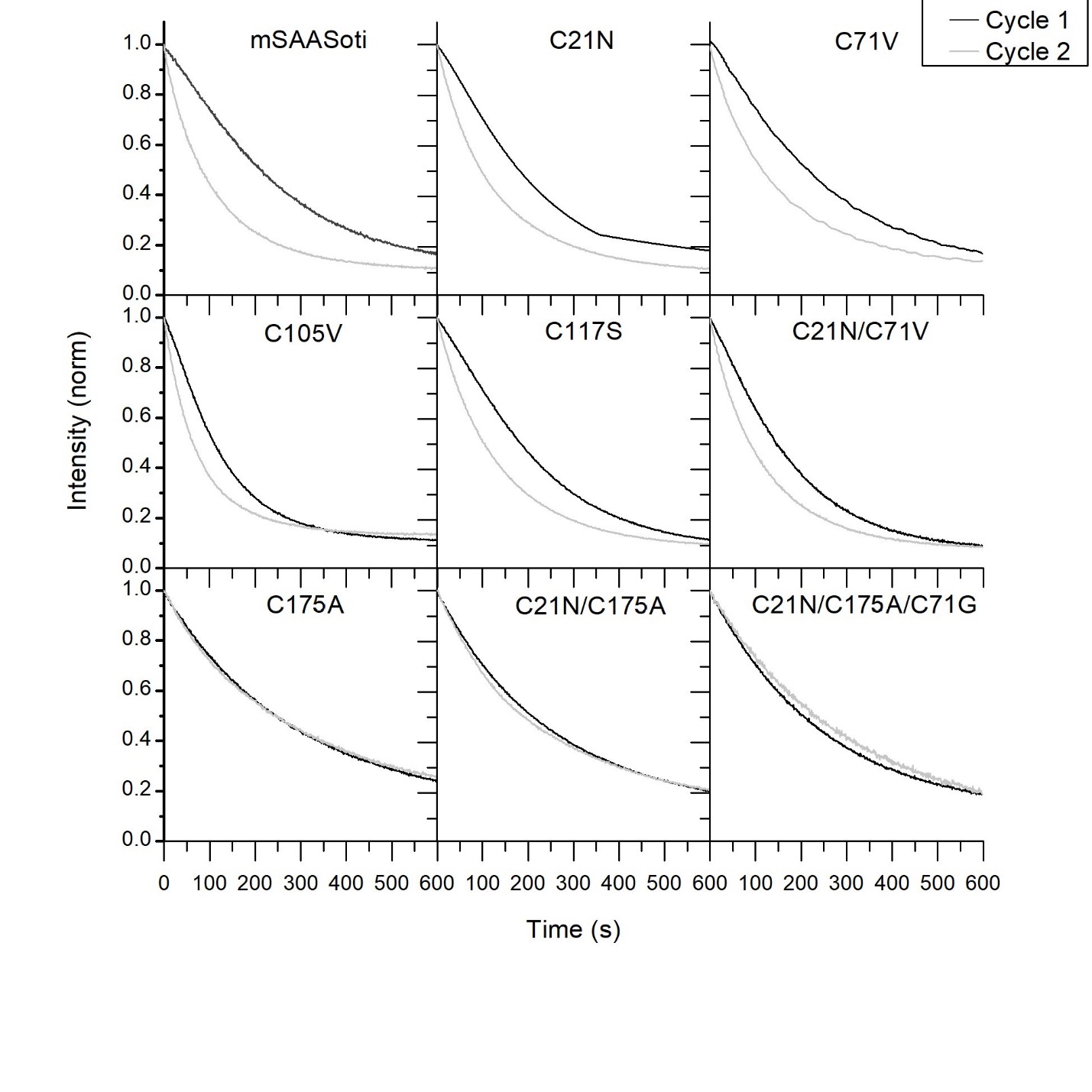


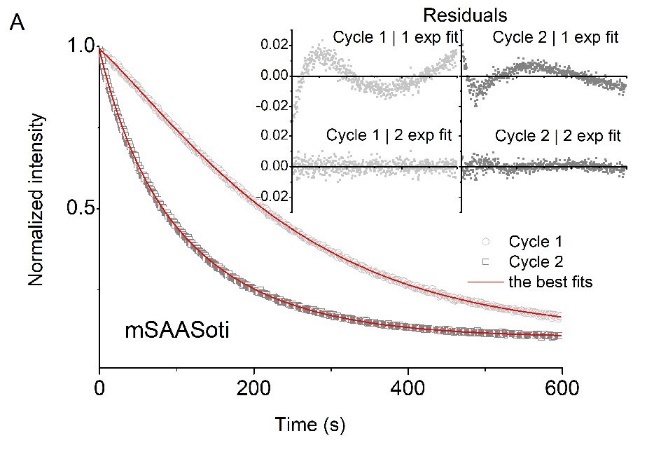

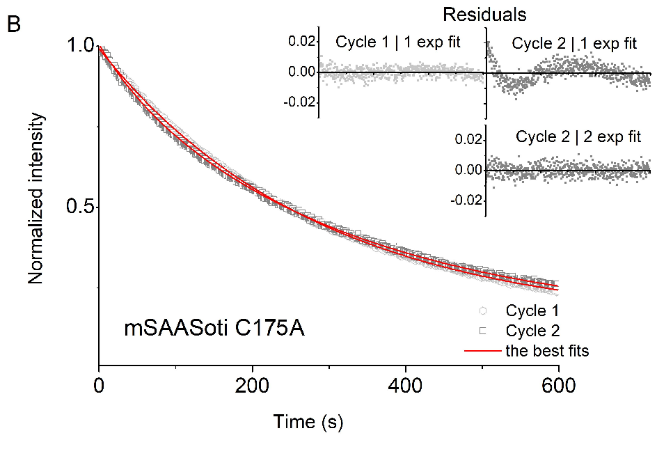


**Figure S3.** (top) Kinetics of green fluorescence on-to-off photoswitching recorded under 470 nm (167 mWt/cm^2^) illumination of different mSAASoti variants (10 μM) in cuvette during 10 min, 1^st^ cycle– black, 2^nd^ cycle – grey. (bottom) photoswitching kinetics (data and fitting) on the example of wild type and C175A SAASoti. Residuals (small figures) as a confirmation of the selected models.

**The probable reaction scheme and the corresponding kinetic equation under 470 nm illumination**

The FPs oxidation at 470 nm irradiation may be described by the next scheme:

$${FP}_{on}\underset{\to}{k1}{FP}_{off}$$

$$and {FP}_{on}\underset{\to}{kox}{FPox}_{on}\underset{\to}{k2} {FPox}_{off}$$

Assuming that oxidation is possible for the on-state only in parallel with photoswitching. Photoswitching is possible for the oxidized FP, too. This form possibly has different ‘brightness’ value at the same wavelength, also because of the spectral shift. The intensity/time dependence is:

$$I_{at X nm}\left( t \right)=i_{1}\cdot\left[ {FP}_{on} \right](t)+i_{2}\cdot\left[ {FPox}_{on} \right](t)$$

Time dependency of $\left[ {FPox}_{on} \right]$ looks like red form FP concentration dependency which is described in the text. The difference is that FPon is consumed in parallel during photoswitching (k1+kox).

$$\left[ {FP}_{on} \right]\left( t \right)={FP}_{on 0}*\exp\left( -(k1+kox)*t \right)$$

$$\left[ {FPox}_{on} \right]\left( t \right)={FP}_{on 0}*\frac{kox}{k2-k1-kox}*\left( \exp\left( -(k1+kox)*t \right)-\exp\left( -k2*t \right) \right)$$

The resulting equation is:

$$I_{at X nm}\left( t \right)={FP}_{on 0}*\left( {(i}_{1}+i_{2}*\frac{kox}{k2-k1-kox} \right)*\exp\left( -\left( k1+kox \right)*t \right)-$$

$$- {FP}_{on 0}*i_{2}*\frac{kox}{k2-k1-kox}*\exp\left( -k2*t \right))$$

Here we can see that the constants determine pre-exponential sign and the intensity flare-up is possible.

**Table S1.** Fitting parameters of photoswitching kinetics (data and fitting) of wild type (A) and C175A (B) SAASoti.

| Fitting parameters | mSAASoti  Cycle 1 | mSAASoti  Cycle 2 | C175A  Cycle 1 | C175A  Cycle 2 |
| --- | --- | --- | --- | --- |
| y0 | 0.113 | 0.103 | 0.180 | 0.134 |
| A1 | 1.472 | 0.714 | 0.803 | 0.777 |
| k1 | 0.0055 | 0.0078 | 0.0038 | 0.0031 |
| A2 | -0.593 | 0.169 |  | 0.094 |
| k2 | 0.0098 | 0.0247 |  | 0.0167 |
| R^2^ | 0.9998 | 0.9998 | 0.9998 | 0.9997 |


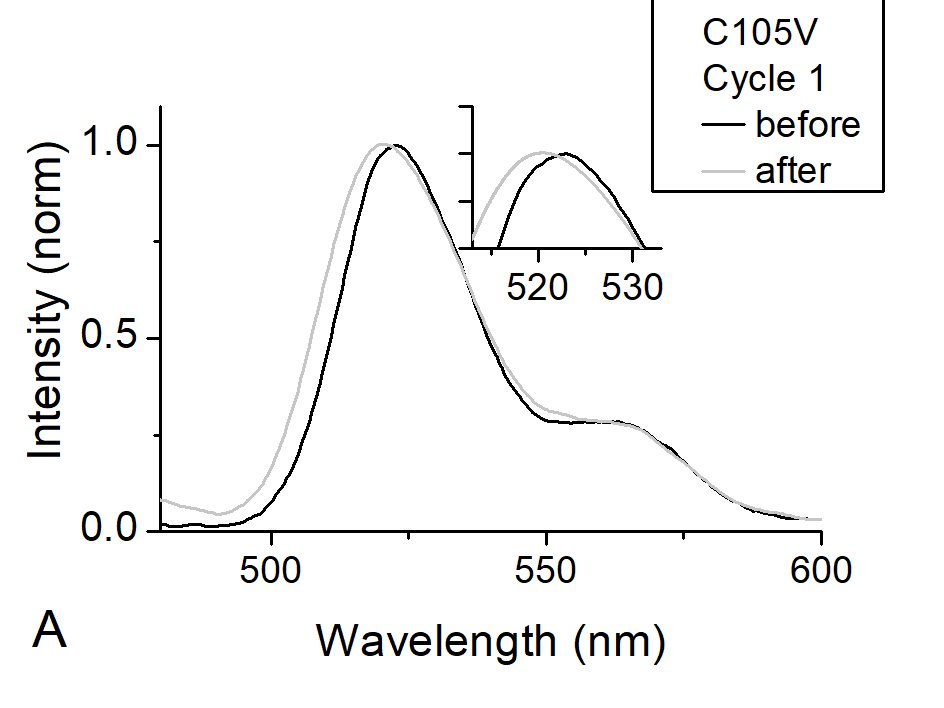

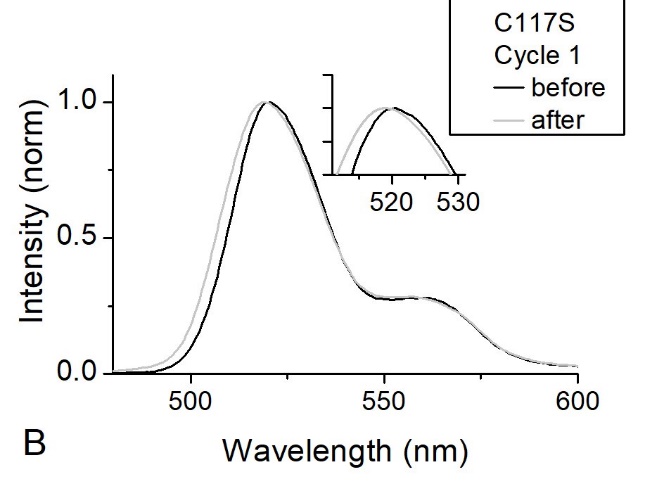

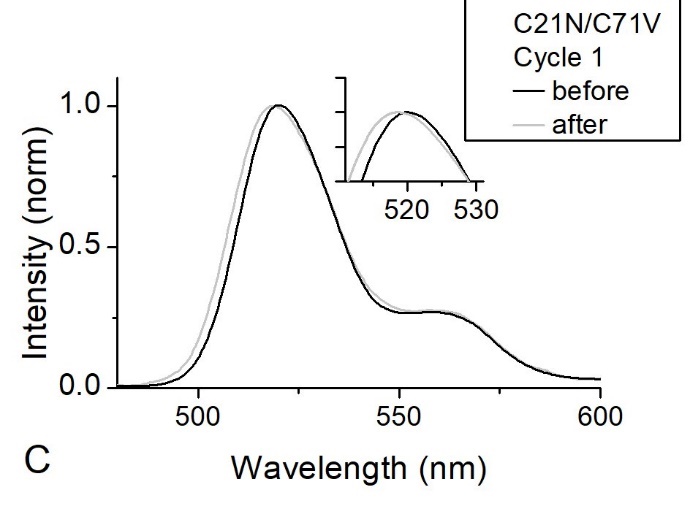

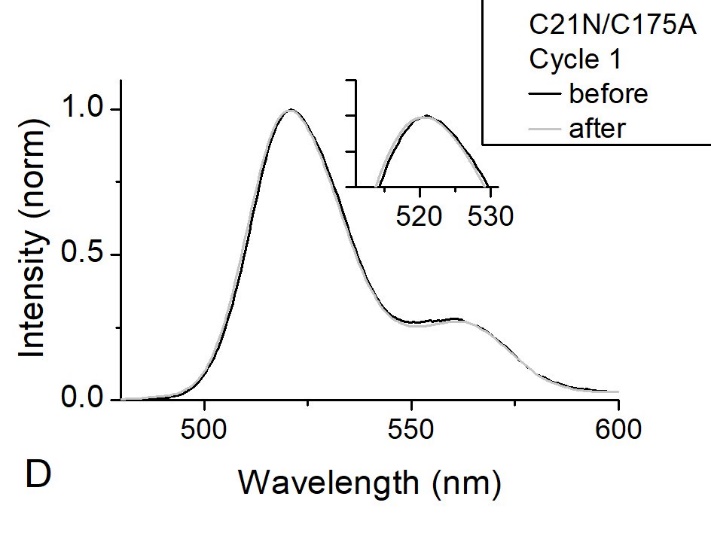

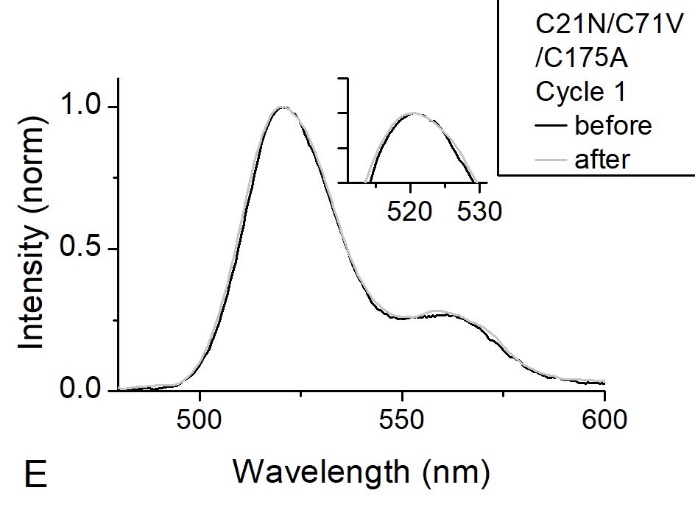


**Figure S4.** mSAASoti variants emission spectra before (black line) and after 500 s of 470 nm light illumination.


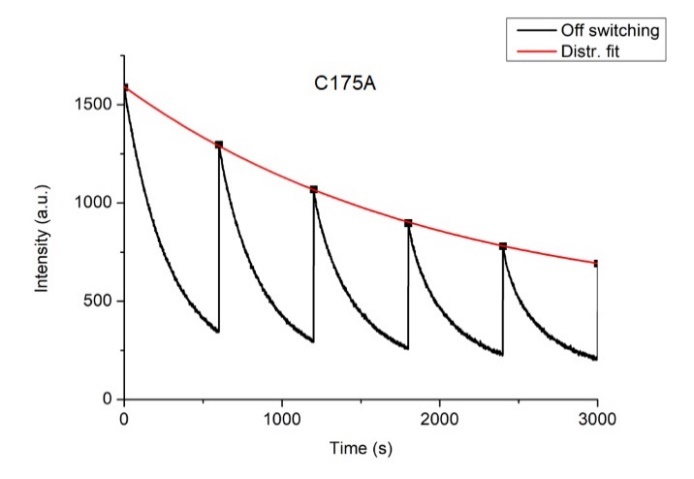

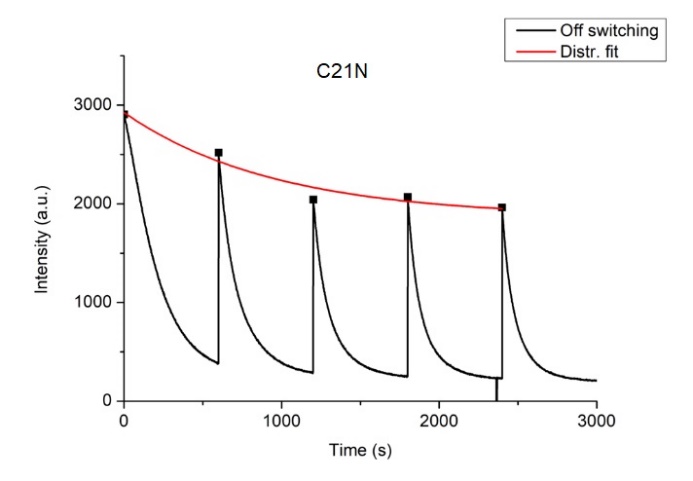

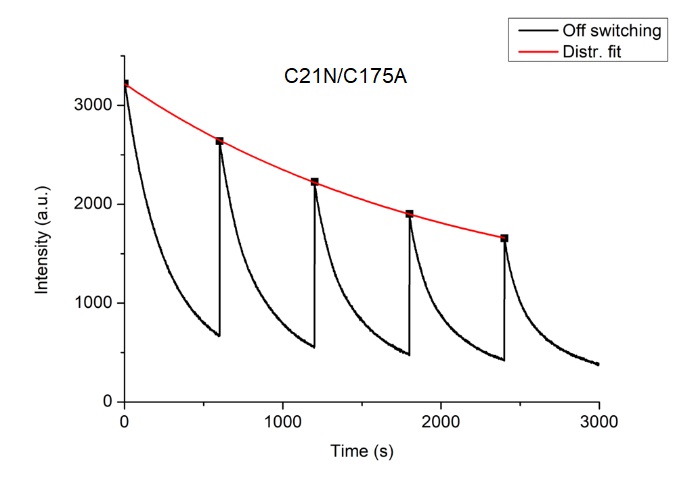

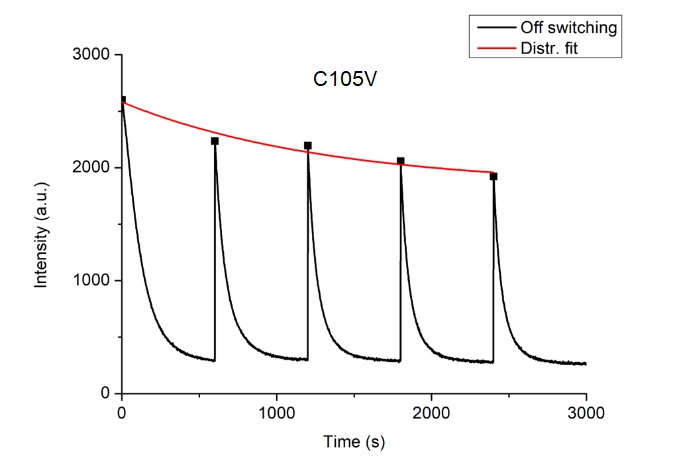

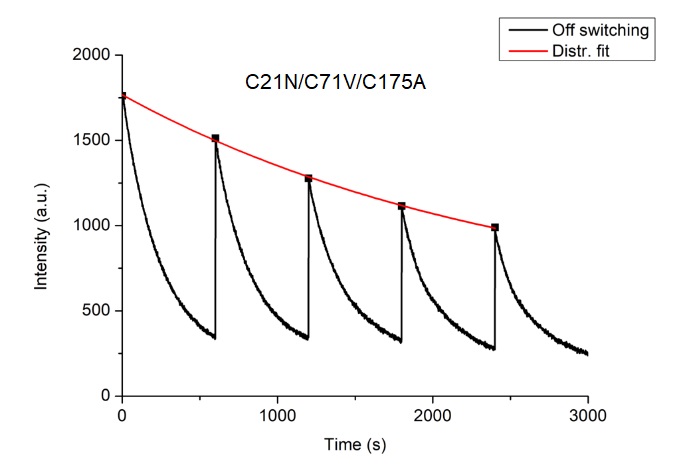

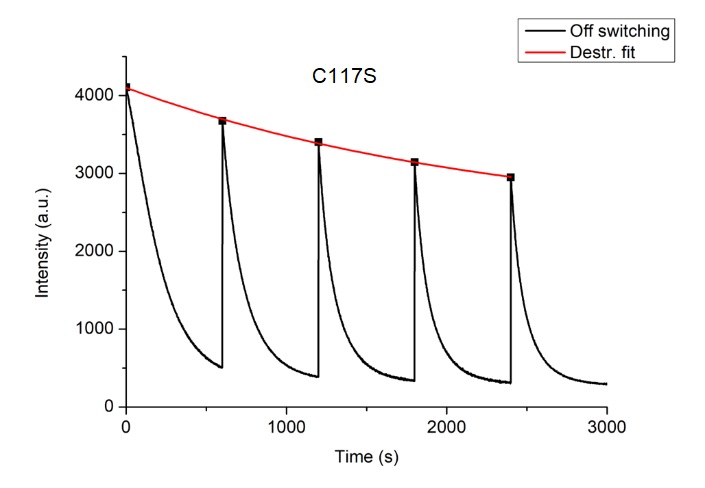


**Figure S5.** Reversible photoswitching of the green SAASoti (10 µM) at 470 nm illumination (167 mW/cm^2^) (10 min) and 400 nm 5.7 mW/cm^2^ (10 s), pH 9.2, 200 mM NaHCO_3_.


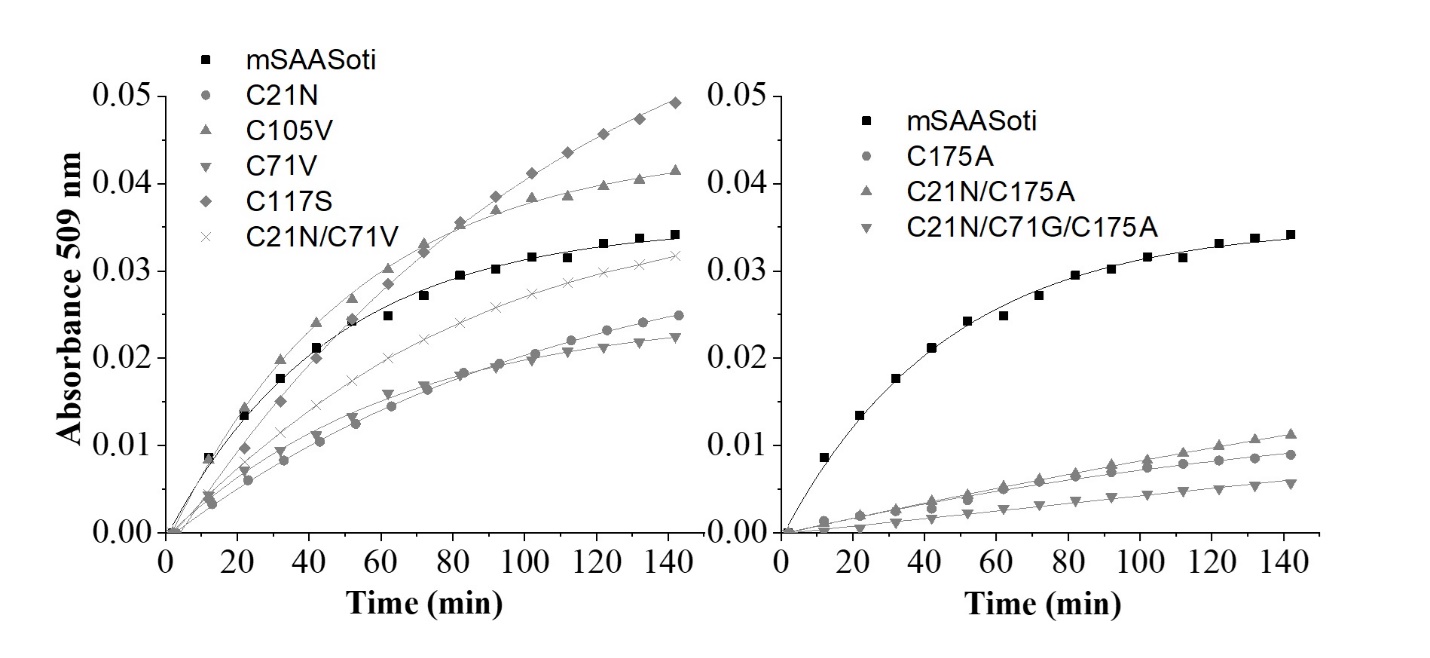


**Figure S6.** Thermal relaxation kinetics measured for different SAASoti mutant forms. The green form (509 nm) absorption recovery in time. Absorption values were normalized to initial A_509_.

**Table S2.** SAASoti variants emission maxima during 470 nm light illumination.

| Peak max | Cycle 1 | | | Cycle 2 | | |
| --- | --- | --- | --- | --- | --- | --- |
|  | 1s  (nm) | 300s (nm) | 500s  (nm) | 1s  (nm) | 300s (nm) | 500s  (nm) |
| C21N | 523.0 | 521.5 | 521.0 | 521.5 | 520.5 | 520.0 |
| C105V | 522.5 | 520.5 | 520.5* | 521.0 | 519.4 | 520.0* |
| C117S | 520.5 | 519.4 | 519.4 | 519.4 | 518.9 | 518.4 |
| C175A | 521.0 | 520.5 | 520.5 | 521.0 | 520.0 | 520.0 |
| C21N/C71V | 519.9 | 518.9 | 518.4* | 518.9 | 518.9 | 518.4* |
| C21N/C175A | 521.0 | 520.5 | 520.5 | 521.0 | 520.5 | 520.5 |
| C21N/C71G/C175A | 521.0 | 520.5 | 520.5* | 521.0 | 520.5 | 520.0 |

**Table S3.** Emission maxima shift during 470 nm light illumination of different mSAASoti variants.

| Δ max | Cycle 1 | | Cycle 2 | | |
| --- | --- | --- | --- | --- | --- |
|  | 300s  (nm) | 500s  (nm) | 1s  (nm) | 300s  (nm) | 500s  (nm) |
| C21N | 1.5 | 2.0 | 1.5 | 2.5 | 3.0 |
| C105V | 2.0 | 2.0 | 1.5 | 3.0 | 2.5 |
| C117S | 1.0 | 1.0 | 1.0 | 1.5 | 2.0 |
| C175A | 0.5 | 0.5 | 0.0 | 1.0 | 1.0 |
| C21N/C71V | 1.0 | 1.5 | 1.0 | 1.0 | 1.0 |
| C21N/C175A | 0.5 | 0.5 | 0.0 | 0.5 | 0.5 |
| C21N/C71G/C175A | 0.5 | 0.5 | 0.0 | 0.5 | 1.0 |

**Table S4.** Emission/excitation wavelength and pKa value of the red form for different mutant SAASoti variants.

|  | λ_ex_/λ_em_, nm | pK_a_ |
| --- | --- | --- |
| **mSAASoti** | 573/579 | 6.6±0.1 |
| **C21N** | 579/590 | 7.5±0.1 |
| **C105V** | 576/589 | 7.1±0.1 |
| **C71V** | 577/590 | 7.0±0.1 |
| **C175A** | 580/587 | 7.8±0.1 |
| **C117S** | 580/590 | 6.7*±*0.1 |
| **C21N/C71V** | 577/589 | 7.0±0.1 |
| **C21N/C175A** | 580/590 | 7.4±0.1 |
| **C21N/C175A/C71G** | 577/589 | 7.2±0.2 |

**Molecular Dynamics Simulations**

For all production runs we calculated RMSD over MD trajectories. Figure S6 demonstrates the RMSD calculated for the backbone atoms relative to the first step of the production run. This proves that the system is equilibrated and the results of the study should be reliable.

**Figure S7.** The RMSD over production MD run calculated for the backbone atoms of SAASoti C175A.
